# Development and evaluation of Tamil Matrix Sentence Test Performance in young adults

**DOI:** 10.1590/2317-1782/20232022263

**Published:** 2023-09-15

**Authors:** Ramya Vaidyanath, Neethi Jesudass, Thaaranya Krishnamoorthy Achari

**Affiliations:** 1 Faculty of Audiology and Speech Language Pathology, Sri Ramachandra Institute of Higher Education and Research - SRIHER (DU) - Chennai, Tamil Nadu, India.

**Keywords:** Matrix Sentence Test, Speech Identification Score, Performance-Intensity Function, Normative, Young Adults

## Abstract

**Purpose:**

The purpose of the study was to develop the Tamil Matrix Sentence Test (TMST) and evaluate the performance of a group of young adults with normal hearing on the developed test. The developed sentences were also administered at varying intensities to obtain a performance-intensity (PI) function.

**Methods:**

A base matrix with 10 sentences containing 5 words each with a total of 50 words was used to develop the TMST. The sentences had a fixed semantic sentence structure of Tamil language in the order of noun, number, adjective, object and verb. The developed test consisted of 30 lists with 10 sentences in each list. The performance of 60 young adults with normal hearing aged 18 to 24 years across the 30 lists were compared for list equivalency. To obtain the PI function the sentences were administered on 20 young adults with normal hearing at intensities from 20 dB HL to 100 dB HL in 10 dB increments. The performance across the intensity levels were compared.

**Results:**

The 30 lists of TMST were found to be acoustically equivalent. However, few lists showed significant difference in the scores obtained on them compared to the rest of the lists. The PI function revealed a saturation in performance beyond 40 dB HL.

**Conclusion:**

From the results it was construed that TMST can be used to evaluate the speech identification abilities of Tamil speaking listeners. Multiple lists offer the advantage of retesting without the influence of practice or listeners memorizing the test material.

## INTRODUCTION

Pure-tone audiometry is often used as a primary procedure to evaluate the type and degree of hearing impairment in an individual. It only provides information on the ability to detect pure-tones and does not represent an individual’s speech perception ability^(1)^. More information about an individual’s ability to perceive speech can be obtained using speech audiometry^(2)^. The most commonly used speech perception measures include the *speech recognition threshold* (SRT) which measures the identification of speech stimuli at threshold level and *speech identification score* which measures the maximum scores obtained by identifying the speech stimuli at supra-threshold level under optimum listening conditions^(3)^. The speech identification measurement can provide useful information about the communication difficulties experienced by listeners with hearing impairment^(4)^. There are several types of stimuli used for speech audiometry such as phonemes, nonsense syllables, monosyllables, spondees and sentences.

Speech identification tests using sentences have been reported to be useful in assessing speech perception abilities^(5)^. Language plays an important role in speech identification testing especially in a multilingual country like India with 22 officially recognized languages^(6)^. However, there are very few test materials developed in Indian languages that use sentences as stimuli and these include the tests developed in Kannada^(7)^, Hindi^(8)^ and Telugu^(9)^.

In Tamil, two tests have been very commonly used for speech identification testing including the Picture Speech Identification Test for children in Tamil^(10)^ and the Phonetically Balanced Test Materials in Tamil Language^(11)^. These tests use bi-syllabic and monosyllabic words as stimuli respectively. To provide more realistic information about speech perception abilities of an individual, sentence tests need to be used. As there are very limited tests available in Tamil, there is a need to develop one to suit the requirements of the Tamil speaking population. The developed test will be useful for testing an individual in their native language to more precisely describe their speech perception abilities. As suggested by Lehiste and Peterson^(12)^ test material in an individual’s native language and dialect should be used to obtain accurate results on speech audiometry testing.

Additionally, the developed test should have multiple lists of equal difficulty and should be quick to administer as well as provide reliable results^(13)^. As Kollmeier and Wesselkamp^(14)^ suggested, the developed sentences should be capable of being used repeatedly with the same subject to evaluate the benefits of the amplification without the effect of familiarity.

Sentences provide more realistic listening situation and have greater face validity than compared to word stimuli for assessing speech recognition ability^(15)^ and it also provides semantics, syntactic and lexical cues^(16)^. Even though sentences have some advantages, there are some factors affecting speech identification testing using sentence stimuli as discussed by Kalikow et al.^(16)^. One such factor is the difficulty in distinguishing whether an individual’s responses were because of good speech recognition skills or because they made good use of linguistic processing skills. Auditory memory has also been reported as one factor affecting the sentence identification scores^(17)^.

To overcome some of the factors noted by Kalikow et al.^(16)^ that affect sentence identification, tests that use sentences constructed from a fixed matrix of words were developed. These sentences have identical grammatical structure and are semantically correct with no redundancy^(18)^. The words from the matrix are combined to form complete sentences^(19-22)^.

According to Hagerman^(20)^, the main purpose of developing matrix sentence test was to assess speech intelligibility in the presence of noise especially to evaluate the benefit of hearing aids in free field and also assess speech discrimination under headphones. They found that multiple lists were required for clinical use. Hence, 13 lists of sentences with noise were developed out of which five sentence lists were evaluated for homogeneity as all the sentences had the same content of sound. The homogeneity of the sentences was evaluated in 20 young normal hearing adults. All the lists were reported to be equally difficult and the performance results fell within the confidence interval except list number one as reported. On observing the learning effect, Hagerman^(20)^ opined that clients could memorise the 50 words and guess the sentence which accounted for 10% chance factor, since the words used in all the lists were the same 50 words. The maximum intelligibility score was obtained at speech levels lower than normal conversational level at constant S/N ratio as reported.

The results reported in the current study are from two experiments carried out. In the first experiment the Tamil matrix sentence test was developed. In the second experiment the developed sentences were presented at varying intensities to obtain the performance-intensity function.

## METHODS

The current study aimed at the development of the Tamil Matrix sentence test and administering the same on a group of young adults with normal hearing. Additionally, the developed sentences were administered at varying intensities to obtain a performance-intensity function for the sentences. Prior to the conduct of the study the methodology was approved by the ethics committee of the institution (CSP/17/APR/56/110). Written informed consent was obtained from all the participants before the initiation of data collection.

### Experiment 1: development of Tamil Matrix sentence test (TMST)

The TMST was developed in the similar format used by Hagerman^(20)^. The sentences followed a fixed structure of Tamil language with nouns, numbers, adjectives, object and verbs. Each word category had 10 alternatives with a total of 50 words forming the base matrix. These words were selected form the vocabulary of children studying in grade I and II. From the 87 words initially selected, 50 words were shortlisted based on familiarity. The familiarity of the words was rated on a 3-point rating scale (0: unfamiliar word; 1: familiar word and 2: highly familiar word) by 20 children studying in grade I and II, 10 parents of children studying in grade I and II and 10 teachers. Words rated as highly familiar and familiar were included to form the base matrix with 50 words forming 10 sentences with 5 words in each. The same has been provided in Chart 1 with the meaning of the selected words in English with their International Phonetic Alphabet (IPA) transcript. These words were selected from the vocabulary of children in order to develop a speech identification test that can be used with large group of population including both children and adults.

**Chart 1:** Matrix of 50 words with their International Phonetic Alphabet (IPA) transcript used for generating the sentences with the meaning of the words in English

**Name**		**Number**		**Adjective**		**Object**		**Verb**	
**Tamil word and IPA transcript**	**Meaning in English**	**Tamil word and IPA transcript**	**Meaning in English**	**Tamil word and IPA transcript**	**Meaning in English**	**Tamil word and IPA transcript**	**Meaning in English**	**Tamil word and IPA transcript**	**Meaning in English**
Arun (əɾun)	Arun	aaru (əːɾu)	Six	azhagana (əɻəgənə)	Beautiful	bomaiyai (boṃməjəi)	Doll	eduthan (Ɛd̪uđən)	Took
baalu (bəːlu)	Baalu	aezhu (Ɛːɻu)	Seven	karuppu (kəɾuppu)	Black	dappava (dəp̣pavə)	Box	kaatnan (kaːtɪnən)	Showed
karthi (kəːrt̪ɪ)	Karthi	anju (əndʓu)	Five	mukkiyamana (muκ̣κɪjəməːnə)	Important	kodaiya (kodəijə)	Umbrella	kettan (kƐṭtan)	Asked
kathir (kədɪr)	Kathir	ettu (ɛʈʈu)	Eight	pazhaya (pəɻəjə)	Old	meenai (miːnəi)	Fish	kuduthan (kuduđđən)	Gave
kumar (kumər)	Kumar	moonu (muːnu)	Three	periya (pɛɾɪjə)	Big	padaththa (pəijəi)	Picture	parthan (pəɾđan)	Seen
naveen (nəviːn)	Naveen	naalu (nəːlu)	Four	sariyana (səɾɪjəːnə)	Correct	paiyai (pəjjəj)	Bag	pirichan (pɪɾɪtjən)	Tore
raamu (rəmu)	Raamu	nooru (nuːɾu)	Hundred	segappu (sɛɡəp̣pu)	Red	penava (pɛnəvə)	Pen	pootan (poːtən)	Thrown
ravi (rəvɪ)	Ravi	ombadhu (onbəđu)	Nine	suththamana (suđ̣đəmɑnə)	Clean	sattaiya (səṭtəijə)	Shirt	thottan (đoṭtən)	Touched
vasu (vəːsu)	Vasu	paththu (pəđ̣đu)	Ten	thappana (đəp̣pənə)	Incorrect	thatta (đəṭtə)	Plate	vanginan (vəngɪnən)	Bought
velu (vƐlu)	Velu	rendu (ɾƐndu)	Two	thevaiyana (đɛvəijanə)	Need	vandiya (vəndɪjə)	Vehicle	vechchan (vƐṭʃtʃan)	Kept

The 10 sentences were audio recorded by a native female Tamil speaker using a Rode unidirectional dynamic microphone held at approximately 10 cm from the mouth. The speaker produced the sentences with normal intonation, constant vocal effort and at regular speaking rate. The sentences were recorded using Nuendo 4 software at a sampling frequency of 44000 Hz and 16-bit resolution. The recorded sentences were then sliced into individual words using Adobe Audition (Version 3) software. The slicing was done at the zero-crossing ensuring that the clarity of the recorded words as well as coarticulatory information were preserved. The loudness of all the recorded words were normalised to ensure all the words were equally loud. The intelligibility of the words was confirmed by a goodness test on 10 young normal hearing adults. These individual words were then concatenated to form sentences using the MATLAB code developed by Gnanateja and Bhattarai^(23)^. A total of 460 possible sentences following the sentence structure of Tamil without repetitions were generated. These sentences were then rated by two speech language pathologists and an audiologist for the appropriateness of the semantic content. They also judged the naturalness of the resynthesized sentences. Three hundred sentences considered as meaningful and naturally sounding by these professionals was used for the final test material. These 300 sentenced were randomly divided into 30 lists with 10 sentences in each list.

Though the test was developed following the design of the matric sentence test as recommended by Hagerman^(20)^ there were a few differences in the administration of the test. The developed test was used for assessing the speech identification performance in quiet rather than the measure of speech recognition threshold (SRT) in noise like the other available matrix sentence tests. The test was administered at a constant intensity 40 dB SL (*re: PTA*) unlike the adaptive intensity used for measuring SRT.

The developed sentences were administered on 60 young adults (30 males & 30 females) aged between 18 and 24 years (mean age = 21.15 years). All the participants had pure-tone thresholds ≤15 dB HL in the octave frequencies from 250 Hz to 8000 Hz for air conduction and from 250 Hz to 4000 Hz for bone conduction. A ‘type A’ tympanogram with presence of ipsilateral and contralateral reflexes indicating normal middle ear functioning was ensured in all participants. The sentences were presented to the participants under headphones at 40 dB SL (*re: PTA*) in one ear that was randomly selected. The participants were instructed to write the sentences they heard. They were encouraged to guess the word in case they were not sure of the response. Short duration breaks were provided during the testing session to sustain the attention of the participants throughout. The responses were scored by giving a score of 1 to each correctly identified word and a score of 0 for the incorrect words. The maximum possible score for each sentence was 5 and for each list was 50. The order of list presentation and the ear of presentation were randomised to avoid any order effect or ear effect respectively.

### Experiment 2: performance-intensity function

For obtaining the performance-intensity function, the recorded sentences were administered on 20 young adults (10 males & 10 females) with normal hearing who were also a part of experiment 1. The sentences were routed through the auxiliary input of the audiometer and presented at intensities of 20, 30, 40, 50, 60, 70, 80, 90 and 100 dB HL. At each intensity level the listeners heard 5 sentences that were randomly selected from the 300 sentences used in experiment 1. The participants heard the sentences only in one ear, with half the participants hearing it in the right while the other half in the left ear. It was ensured that the ear of presentation and the order of the intensity presentation was randomised to avoid any ear order effect. The participants were instructed to write down the sentences heard. From the responses obtained, percentage of words correctly identified at each level was calculated. Further, the performance across the intensity levels were compared.

### Statistical analysis

Descriptive and analytical statistics was done using IBM SPSS software (Version 18). Before the analytical statistics was done the Shapiro-Wilk test of normality was carried out. As the data did not show a normal distribution, non-parametric tests were used. To compare the performance across the 30 lists of sentences, a Friedman test followed by Wilcoxon pairwise comparison was done.

## RESULTS

The findings of the two experiments of the study are provided separately. In the first experiment, the performance of the 60 participants on the 30 lists of TMST are provided. In the second experiment, the results of the performance-intensity function obtained from 20 listeners are provided.

### Experiment 1

**Figure 1:**
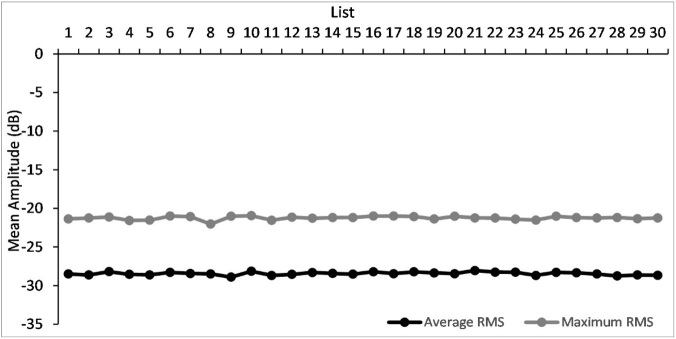
Mean average and maximum Root Mean Square (RMS) amplitude of the 30 lists of sentences of Tamil Matric sentence test

The Tamil matrix test developed had 30 lists of 10 sentences each. The average, minimum and maximum RMS of the 30 lists is provided in Figure 1. To ensure the amplitude of all the sentences were equivalent, these measures were compared. As average RMS showed normal distribution repeated measure ANOVA was used for comparison while, Friedman test was used for comparison of minimum and maximum RMS across the 30 lists. The results revealed that a significant difference was not obtained between the amplitude measures of average RMS (F(29, 261) = 0.92, *p* > .05), minimum RMS [χ^2^(2) = 36.27, *p* > .05] and maximum RMS [χ^2^(2) = 22.99, *p* > .05] of the sentences across the 30 lists.

The mean, median and the standard deviations of the scores obtained by the 60 young listeners are provided in Table 1. It can be observed from the table that the scores across the 30 lists varied only marginally. A Friedman test was done to evaluate if this was significant.

**Table 1:** Mean, median and standard deviation (SD) of scores obtained by 60 young adults across 30 lists of TMST

List No.	Score		
	Mean	Median	SD
1	49.65	50.00	.82
2	49.77	50.00	.62
3	49.70	50.00	.72
4	49.75	50.00	.68
5	49.67	50.00	.73
6	49.68	50.00	.77
7	49.58	50.00	.89
8	49.43	50.00	1.03
9	49.57	50.00	.96
10	49.88	50.00	.37
11	49.62	50.00	.78
12	49.58	50.00	.94
13	49.57	50.00	.83
14	49.82	50.00	.62
15	49.75	50.00	.51
16	49.70	50.00	.83
17	49.75	50.00	.47
18	49.68	50.00	.87
19	49.68	50.00	.65
20	49.67	50.00	.63
21	49.55	50.00	.91
22	49.63	50.00	.76
23	49.57	50.00	.92
24	49.70	50.00	.65
25	49.47	50.00	1.05
26	49.42	50.00	1.12
27	49.77	50.00	.62
28	49.60	50.00	.91
29	49.83	50.00	.61
30	49.68	50.00	.6

Maximum score = 50

Before the comparison of the scores across the lists, the scores of the male and female participants were compared using Mann Whitney test statistic (U). It was found that except list 5 and 26, the scores of the two groups were not significantly different (Table 2). Hence, the data from the two genders was combined to compare the performance of the right and left ear. It was observed that scores of the two ears were similar across all the 30 lists of TMST (Table 2).

**Table 2:** Mann Whitney test statistic (U) comparing performance of males and females as well as the scores of two ears in 30 lists of TMST

	Gender comparison		Ear comparison	
List No.	U	p	U	p
1	401.50	.32	407.50	.39
2	438.00	.79	373.00	.09
3	435.00	.76	435.00	.76
4	432.50	.68	434.50	.72
5	304.00	.003**	367.50	.10
6	385.00	.19	391.50	.24
7	356.50	.07	392.50	.28
8	350.50	.08	441.50	.89
9	432.50	.73	437.50	.81
10	448.50	.97	418.50	.38
11	424.00	.62	422.00	.6
12	356.00	.06	423.00	.59
13	415.50	.51	367.00	.12
14	435.00	.69	403.00	.22
15	435.50	.76	428.50	.67
16	426.00	.58	446.50	.94
17	417.00	.51	439.00	.83
18	389.50	.17	418.50	.48
19	395.00	.27	415.50	.5
20	424.50	.62	389.00	.25
21	444.50	.92	430.50	.72
22	386.00	.21	440.00	.85
23	402.50	.35	436.50	.80
24	431.00	.69	424.00	.6
25	409.50	.45	344.50	.05
26	339.50	.04*	448.50	.98
27	403.00	.26	435.00	.73
28	416.50	.50	418.50	.53
29	420.50	.40	391.50	.1
30	380.00	.17	415.00	.50

** p < .01

* p < .05

Further, comparisons of the scores using the Friedman test revealed that there was a significant difference in the total score obtained across the 30 lists of TMST (χ^2^(29) = 63.61, p < .001). Pairwise comparison was carried out using Wilcoxon signed rank test to reveal which lists were different from each other. The results of the pairwise comparison is provided in Appendix A. Based on these pair-wise comparisons, List 8, 10, 14, 24, 25 and 29 were found to differ significantly from most of the other lists.

### Experiment 2

**Figure 2:**
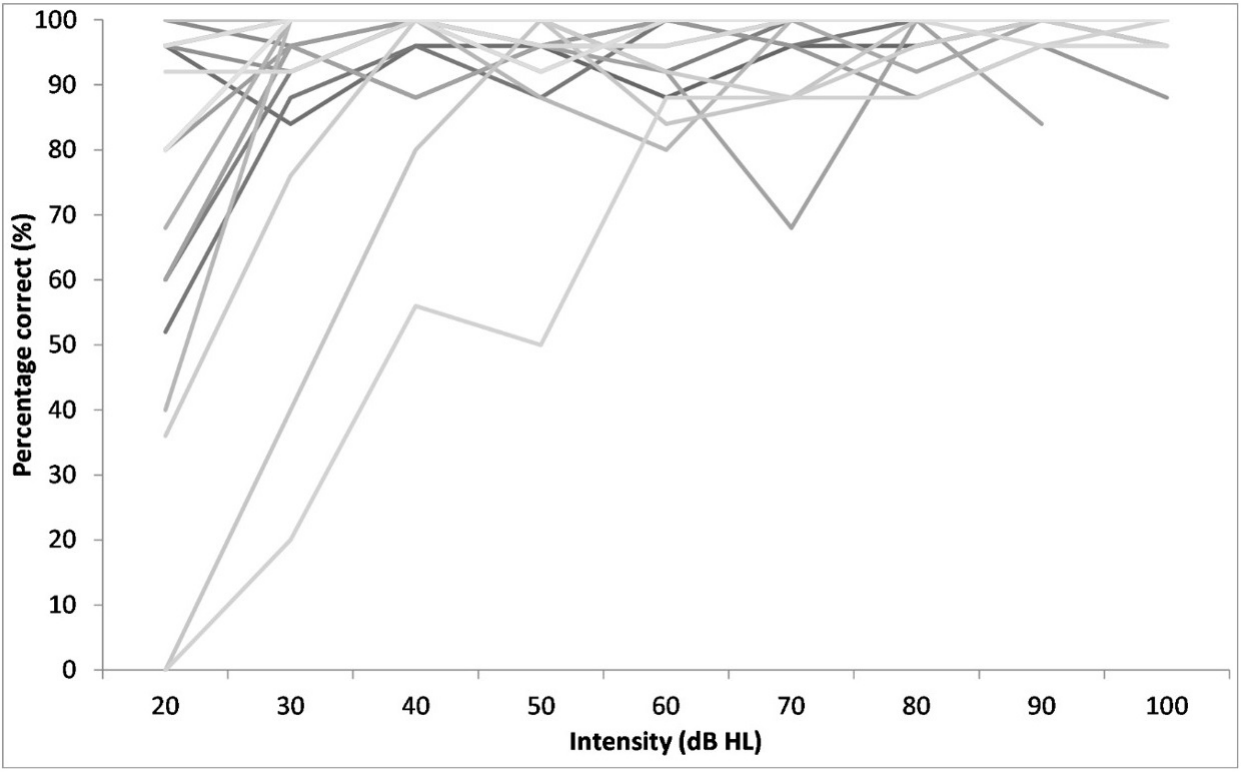
Performance-intensity function of 20 participants for the TMST sentences

In the second experiment, the percentage recognition of the sentences at increasing intensities were obtained. It can be observed from Figure 2 that as the intensity increased the performance improved. A Friedman test with intensity and performance as factors revealed a significant effect of intensity (χ^2^(8) = 51.26, p < .001) on performance. Further, pairwise comparisons of the performance across the intensities were carried out using Wilcoxon test (Table 3). It can be observed that the performance at the lowest intensity tested (20 dB HL) was significantly poorer compared to the rest of the intensities. It can also be observed from the Figure 2 that significant improvement in performance was not observed with increase in intensity beyond 40 dB HL.

**Table 3:** Wilcoxon signed rank test statistic (z) and significance (p) for pair-wise comparison of intensities

			30	40	50	60	70	80	90	100
20		z	-3.21	-3.63	-3.27	-3.74	-3.63	-3.38	-3.63	-3.21
		*p*	.001**	.000***	.001**	.000***	.000***	.001**	.000***	.001**
30		z		-2.23	-1.19	-1.43	-2.13	-2.21	-2.39	-1.58
		*p*		.026*	.23	.15	.03*	.03*	.017*	.11
40		z			-1.03	-.55	-.25	-1.13	-1.29	-.26
		*p*			.30	.58	.8	.26	.2	.8
50		z				-.07	-1.04	-1.88	-2.17	-1.23
		*p*				.94	.30	.06	.03*	.22
60		z					-.91	-1.57	-2.06	-1.13
		*p*					.36	.12	.04*	.26
70		z						-.95	-2.05	-.35
		*p*						.34	.041*	.72
80	z								-.86	-.13
	*p*								.39	.89
90	z									-1.72
	*p*									.08

*** p < .001

** p < .01

* p < .05

The results of the two experiments carried out are discussed separately. Experiment 1 revealed that even though the mean scores across the 30 lists were similar, differences in the performance did exist on the lists. In Experiment 2, the positive effect of intensity on performance was observed up to 40 dB HL.

From the results of the two experiments, it can be observed that though the 30 lists of TMST were equivalent acoustically, the performance on them varied. As six lists were found to be significantly different to the others it is suggested these not be used even though the mean performance of these were similar to the others.

## DISCUSSION

Two experiments were carried out as part of the current study. The first included the development of TMST and evaluating the performance of 60 young adults on it. In the second experiment, the performance of the young listeners at increasing intensity levels was assessed.

The material for TMST was chosen based on the familiarity of children aged 6 to 7 years. Hence, the test can be used for evaluating speech perception abilities of children 6 years and older. However, a normative may be established prior to its use with children. Many studies have recommended that the test material used for speech perception testing be familiar to the listener^(15,24)^ and even when the participants are familiar with more than one language, the test material should be presented in the native language of the listener^(25)^.

Additionally, it was also ensured that the words used in the test were similar across the different dialects of Tamil language spoken in Tamil Nadu (a southern state of India). Therefore, the test can be used throughout the state without the dialectal influences.

During the development of the test material of TMST, the words were spliced from the spoken sentences. This ensured that the co-articulatory cues were retained and when the words were concatenated, they sounded more natural.

In the first experiment, it was found that all the 30 lists had similar amplitude as the minimum and maximum RMS as well as the average RMS across the lists were not significantly different (Figure 1). This indicates that the amplitude variation among the sentences were similar. Further, it was observed that the mean and median score obtained were also similar across these lists. Considering that the sentences had minimal influence of the loudness variation (equally loud), the constant performance across the lists can be expected.

It was further observed that even though the mean and median scores across the lists were similar, performance on some lists (8, 10, 14, 24, 25 & 29) were found be different from the rest of the lists. A closer observation of the average and maximum RMS of these lists showed that list 8 and 24 had slightly lower RMS measures compared to the rest of the lists.

The two genders also showed similar performance on the lists. Additionally, the scores on the 30 lists remained same irrespective of the ear of presentation. Hence, it can be construed that the developed test can be used for all individuals without the influence of gender or the ear of stimulation.

The results of the second experiment indicates that the performance of young adults improved with increase in intensity till about 30 to 40 dB HL and saturated thereafter. Based on the findings it can be concluded that these matrix sentences in Tamil can be used even at higher intensities without affecting the performance. This finding is of clinical importance especially while using these sentences in evaluating performance with hearing aids in individuals with hearing impairment.

The developed matrix test has advantage of being able to test an individual multiple times without the individual being able to memorize the sentences. This is important while testing using multiple hearing aids/program settings or research evaluating speech perception abilities in multiple test conditions. These advantages were also noted by Jansen et al.^(26)^.

The current study was carried out in young adults. The utility of the test in evaluating the speech perception abilities in children and older adults may be further tested. Additionally, the effectiveness of the test material in assessing speech perception in the presence of noise and in those with hearing impairment may also be evaluated.

## CONCLUSION

The TMST with its 30 sentence lists can be used clinically to evaluate the speech identification ability of an individual. It was observed that the intensity had to be at least 40 dB HL to achieve 100% identification. There after a minimal change in the performance was observed. Based on the results it was concluded that the Matrix sentences could be useful in differentiating individuals with good and poor speech identification at higher intensities. The performance of the young adults in the current study can be used as normative for evaluating whether an individual’s speech identification is normal. These sentences can be used to evaluate individuals with hearing aids which may require the sentences to be perceived at higher intensity levels. Further, the words used in the construction of the base matrix were taken from the vocabulary of 6 to 7 year old children, hence the test may be applied in these population as well.

## Appendix A

**Table:** Pairwise comparison of the performance of young adults on the 30 lists using Wilcoxon signed rank test (shaded blocks indicate comparisons that are significant

List	1	2	3	4	5	6	7	8	9	10	11	12	13	14	15	16	17	18	19	20	21	22	23	24	25	26	27	28	29	30
1		-.84	-.33	-.95	.00	-.41	-1.92	-.85	-.51	-2.4	-.47	-.49	-1.18	-1.29	-.80	-.45	-1.11	-.21	-.26	-.26	-1.36	-.17	-1.21	-.28	-2.14	-2.24	-1.01	-.68	-1.98	-.26
2			-1.0	.00	-1.06	-1.09	-2.05	-2.84	-1.98	-1.6	-1.54	-2.08	-1.93	-.83	-.39	-.27	-.41	-1.06	-1.03	-1.26	-2.28	-1.71	-1.46	-.85	-2.10	-2.33	.00	-1.8	-1.07	-.99
3				-.85	-.47	-.23	-1.19	-2.30	-1.59	-2.36	-.75	-1.27	-1.28	-1.29	-.41	-.33	-.45	-.25	-.22	-.57	-1.9c	-.94	-1.09	-.17	-1.82	-2.17	-.94	-1.18	-2.31	-.2
4					-1.14	-.89	-1.55	-2.40	-2.00	-1.81	-1.32	-1.68	-1.73	-.97	-.12	-.27	-.07	-.92	-1.04	-1.01	-2.08	-1.43	-1.75	-.62	-2.28	-2.27	-.06	-1.44	-1.03	-.89
5						-.21	-.93	-2.18	-1.29	-2.67	-.40	-1.05	-1.02	-1.43	-.82	-.38	-.98	.00	-.17	.00	-1.33	-.39	-.83	-.45	-2.07	-2.33	-1.32	-.79	-2.36	-.24
6							-1.35	-2.56	-1.12	-2.04	-.62	-.98	-1.02	-1.62	-.41	-.18	-.39	-.02	-.16	-.59	-1.71	-.63	-.98	-.16	-1.71	-2.14	-.91	-1.11	-2.5	-.24
7								-1.71	-.30	-2.69	-.47	-.17	-.23	-2.4	-1.54	-1.01	-1.57	-.91	-.82	-.66	-.40	-.56	-.06	-.94	-.85	-1.21	-1.82	-.18	-2.97	-1.21
8									-1.07	-3.32	-1.85	-1.33	-.99	-2.88	-2.27	-1.96	-2.4	-2.06	-1.87	-1.88	-1.61	-1.95	-1.16	-2.11	-.16	-.02	-2.74	-1.88	-3.62	-2.56
9										-2.94	-.44	-.03	-.07	-2.28	-1.38	-.99	-1.55	-1.19	-1.13	-.78	-.15	-.59	.00	-1.06	-.90	-1.11	-1.88	-.13	-2.88	-.93
10											-2.56	-2.69	-3.35	-.76	-2.00	-1.81	-1.89	-2.14	-2.83	-2.55	-3.14	-2.72	-2.81	-2.3	-3.25	-3.32	-1.93	-2.62	-.58	-2.56
11												-.25	-.61	-2.46	-1.28	-.95	-1.53	-.57	-.73	-.40	-1.00	-.21	-.41	-.64	-1.05	-1.47	-1.35	-.21	-2.36	-.66
12													-.16	-2.4	-1.34	-1.01	-1.66	-.94	-1.06	-.64	-.35	-.41	-.07	-.91	-1.10	-1.21	-1.74	-.15	-2.84	-.97
13						p <.05								-2.63	-1.71	-1.73	-1.95	-.94	-1.33	-1.00	-.3	-.66	-.13	-1.24	-.52	-1.11	-1.87	-.36	-2.62	-1.07
14						p <.01									-.96	-.81	-1.00	-1.5	-1.54	-1.6^a^	-2.62	-1.9	-2.01	-1.28	-2.62	-2.43	-.74	-2.19	-.25	-1.35
15						p <.001										-.08	.00	-.53	-1.00	-.81	-1.74	-1.09	-1.47	-.63	-1.97	-2.18	-.17	-1.24	-1.11	-.85
16																	-.3	-.29	-.38	-.30	-1.56	-.66	-1.04	-.21	-2.33	-1.86	-.44	-1.05	-1.35	-.33
17																		-.58	-1.19	-1.09	-1.72	-1.23	-1.8	-.66	-2.30	-2.51	-.17	-1.37	-1.23	-.89
18																			-.12	-.47	-1.54	-.59	-.97	-.10	-1.61	-1.97	-.91	-.88	-1.98	-.12
19																				-.22	-1.24	-.58	-1.33	-.17	-2.05	-2.10	-1.08	-.78	-1.77	-.02
20																					-.96	-.21	-1.10	-.45	-1.98	-2.14	-.92	-.40	-2.1	-.22
21																						-.97	-.36	-1.46	-.53	-1.30	-2.12	-.77	-3.22	-1.53
22																							-.58	-.48	-1.28	-1.43	-1.55	-.41	-2.56	-.63
23																								-1.22	-.92	-1.44	-1.61	-.54	-2.35	-1.05
24																									-2.0	-2.57	-.59	-.96	-1.61	-.24
25																										-.37	-2.15	-1.1	-2.86	-1.97
26																											-2.57	-1.82	-2.95	-2.34
27																												-1.56	-1.03	-1.03
28																													-2.81	-.96
29																														-1.94
